# Surgical site infection in high-energy peri-articular tibia fractures with intra-wound vancomycin powder: a retrospective pilot study

**DOI:** 10.1007/s10195-015-0352-0

**Published:** 2015-05-10

**Authors:** Keerat Singh, Jennifer M. Bauer, Gregory Y. LaChaud, Jesse E. Bible, Hassan R. Mir

**Affiliations:** Division of Orthopaedic Trauma, Vanderbilt Orthopaedic Institute, Suite 4200 MCE, South Tower, Nashville, TN 37232 USA

**Keywords:** Tibial plateau, Tibial plafond, Pilon, Vancomycin powder, Infection

## Abstract

**Background:**

Surgical site infections (SSI) continue to be a significant source of morbidity despite the introduction of perioperative intravenous antibiotics. Our objective was to assess the efficacy of local vancomycin powder on lowering deep SSI rates in high-energy tibial plateau and pilon fractures.

**Materials and methods:**

A retrospective review of all tibial plateau and pilon fractures treated in 2012 at our level I trauma center identified 222 patients. Of these, 107 patients sustained high-energy injuries that required staged fixation, and 93 had minimum 6 month follow-up. Ten patients received 1 gram vancomycin powder directly into the surgical wound at the time of definitive fixation, and the remaining 83 patients served as controls. SSI was defined according to criteria from the Centers for Disease Control. Demographic data, patient comorbidities, injury and treatment details, and infection details were recorded. Descriptive and comparative statistics were performed.

**Results:**

Amongst the vancomycin powder group, 1 patient (10 %) developed a deep SSI; in the control group, 14 (16.7 %) developed deep SSI. The rate of deep SSI between the groups was not statistically significantly different (*P* = 1.0). The groups were statistically similar with regard to injuries, treatment, comorbidities, and infectious outcomes (*P* values range = 0.06–1.0).

**Conclusions:**

The application of local vancomycin powder into surgical wounds of high-energy tibial plateau and pilon fractures did not reduce the rate of deep SSI in this retrospective review. There is a need to find effective, cheap, and widely available methods for prevention of SSI. Basic science and larger prospective clinical studies are needed to further delineate the role of local vancomycin powder as a modality to reduce deep SSI in extremity trauma.

*Level of evidence* Level III, therapeutic.

## Introduction

Infection is a well-known complication of operative fixation of extremity fractures. Such infections have a significant effect on patients and often require repeat surgery, prolonged systemic antibiotic use and lead to delay in fracture healing and rehabilitation. The risk of mortality doubles in a patient with a surgical site infection (SSI) and the cost of care increases significantly [1]. Risk factors for post-traumatic infection include open fractures, smoking, alcoholism, sustained intensive care unit stay, inadequate debridement at the fracture site and malnutrition [2, 3]. In a patient sustaining high-energy trauma, immune system dysfunction results, as evidenced by decreased ability of polymorphonuclear leukocytes (PMNs) in chemotaxis, decreased superoxide formation and decreased microbial elimination [4]. In the setting of fractures, bone that is devoid of periosteum and other surrounding devitalized tissue serve as an ideal medium for bacterial colonization and replication. Introduction of hardware to appropriately manage a fracture becomes yet another suitable medium for bacterial proliferation given that most metals are electrochemically active and promote molecular adhesion [5].

Some authors have reported infection rates of 14–60 % in high-energy lower extremity fractures [6–11]. Systemic antibiotics reduce surgical site infections, but they are limited by the risk of toxicity. Additionally, traumatic tissues have a compromised blood supply, further limiting the effectiveness of systemic antibiotics. Local antibiotics avert this by sterilizing the wound and preventing the development of a biofilm. The development of a complex glycocalyx biofilm around bacterial colonies greatly impedes the delivery of systemic antibacterial agents and allows bacteria to become more virulent than in their non-adherent state [12, 13].

Administration of perioperative, intravenous antibiotics prior to making a surgical incision has become standard of care and has been effective at reducing surgical infections [14, 15]. Vancomycin is a glycopeptide that inhibits bacterial cell wall synthesis and is used mostly in the prophylaxis and treatment of Gram-positive bacteria. Topical vancomycin offers advantages over systemic administration because higher local concentrations can be achieved and directed to the site of need. Additionally, organisms that may be otherwise resistant to a given systemic therapeutic concentration may be sensitive to the local concentrations [16]. Local antibiotics are commonly delivered via cement beads and spacers. However, these are not always practical, as they require space for placement and necessitate late removal.

Topical vancomycin powder has become popular over the last decade as its efficacy in spine surgery has been demonstrated. A meta-analysis pooling 5888 spine patients that evaluated the effectiveness of topical vancomycin powder at preventing SSI and deep incisional infections showed a significant protective effect of vancomycin powder [17]. Despite extensive research into vancomycin powder efficacy in the spine literature and some work in the field of vascular surgery, no literature exists on vancomycin powder use in prevention of surgical wound infection in extremity injuries.

The objective of our retrospective review was to assess the efficacy of intraoperative vancomycin powder administration on preventing deep SSI in high-energy lower extremity trauma of the tibial plateau and pilon. We hypothesized that the use of intrawound vancomycin powder would reduce the incidence of deep SSI in the treatment group as compared to well-matched controls.

## Materials and methods

### Inclusion/exclusion criteria, treatment, and data collection

The study protocol was approved by the institutional review board at the participating level I trauma center. Inclusion criteria consisted of patients older than 18 years of age who had undergone staged operative fixation of their high-energy tibial plateau or pilon injury. Staged treatment (external fixation, limited internal fixation, or splinting) was defined as definitive internal fixation at a minimum of 5 days post-injury after swelling had resolved. Exclusion criteria consisted of patients with follow-up less than 6 months.

A total of 222 adult patients were found in the institutional database to have sustained tibial plateau and pilon fractures between 1 January 2012 and 31 December 2012. Of these injuries, 50 were treated non-operatively, and 65 did not require staged fixation. The remainder of these injuries (*n* = 107) were treated by the six board-certified orthopaedic trauma surgeons at our institution. Of the 107 patients, 14 had a follow-up time of less than 6 months, leaving 93 patients in the final analysis group. Charts and operative reports of all injuries were reviewed, and ten patients (10.8 %) were documented to have received 1 gram vancomycin powder (Hospira, Lake Forest, IL) directly into the surgical wound at time of definitive fixation at the discretion of the treating surgeon. The remaining 83 patients served as the control group.

All patients received standard systemic antibiotic prophylaxis consisting of 1 g IV cefazolin within 1 h of surgical incision, followed by 1 g IV cefazolin every 8 h for 24 h postoperatively. If the patient was allergic to penicillin, 900 mg IV clindamycin was used instead. Patient demographics (age and gender), injury type (location, open versus closed), smoking status, presence of diabetes, staged treatment with external fixation, presence of single or dual incision, presence of concomitant compartment syndrome and time (in days) from presentation to definitive surgery were all recorded. All patients records were evaluated for signs of SSI for a minimum of 6 months post-operatively. A deep SSI was defined as one requiring operative irrigation and debridement. Wound site erythema without the presence of fluctuance, drainage or purulence was defined as a superficial SSI. Amongst the deep SSI patients, intra-operative microbiological data was also collected.

### Statistics

Using SPSS 22.0 software, dichotomous data was compared using Fisher’s exact tests, while independent *t* tests and Mann–Whitney *U* tests were used for comparisons of parametric and non-parametric data, respectively. Statistical significance was set at *P* < 0.05.

### Outcome measure

The primary outcome measure was the occurrence of deep SSI.

## Results

Demographic parameters did not differ statistically between the two groups. The average age of the vancomycin-treated group was 55 years (range 38–73 years), and the control age was 46 years (range 17–82 years), *P* = 0.064. Of the 10 vancomycin-treated patients, 6 were male (60 %) and 4 were female (40 %), and of the 83 control patients, 55 (54 %) were male and 38 (46 %) were female (*P* = 1.0). Thirty-six (43 %) out of the 83 control subjects were smokers and 2 (20 %) of the vancomycin-treated were smokers (*P* = 0.191). Six (7 %) of the 83 control patients were diabetic whereas no patient in the vancomycin-treated group had diabetes (*p* = 1.0). Of the ten vancomycin-treated patients, five (50 %) sustained a tibial plateau injury and five (50 %) sustained a tibial plafond (pilon) injury. Forty-six injuries in the control group were pilon injuries and 37 were plateau injuries. Comparison of injury type amongst the two groups was not statistically significant (*P* = 0.751). A total of 3 injuries (30 %), all pilon, were open injuries in the vancomycin-treated group, and 23 (28 %) injuries were open in the control group (*P* = 1.0). Patient characteristics and outcomes for the treatment and control groups are presented in Table 1.

**Table 1:** Patient demographics, injury, treatment characteristics and rate of infection

	Control	Treatment	*P* value
Total (*n*)	83	10	
Average age (years)	46	55	0.06
Gender			
M	45	6	1.00
F	38	4	1.00
Smoker *n* (%)	36 (43 %)	2 (20 %)	0.19
Diabetic *n* (%)	6 (7 %)	0 (0 %)	1.00
Pilon injuries *n* (%)	46 (55 %)	5 (50 %)	0.75
Plateau injuries *n* (%)	37 (45 %)	5 (50 %)	0.75
Open injuries *n* (%)	23 (28 %)	3 (30 %)	1.00
Staged treatment with external-fixation *n* (%)	74 (89 %)	9 (90 %)	1.00
Time to definitive fixation (days)	15.6	19.8	0.15
Single incision *n* (%)	34 (41 %)	4 (40 %)	1.00
Dual incision *n* (%)	49 (59 %)	6 (60 %)	1.00
Presence of compartment syndrome *n* (%)	7 (8 %)	1 (10 %)	1.00
Superficial infection *n* (%)	7 (8 %)	0 (0 %)	1.00
Deep infection *n* (%)	14 (16.8 %)	1 (10 %)	1.00

Amongst the vancomycin-treated group, one patient (10 %) developed a deep SSI. In the control group of 83 patients, 14 patients (16.8 %) developed deep SSI. The rate of deep SSI was not statistically different between the two groups (*P* = 1.0). Seven (50 %) of the 14 patients in the control group that developed a deep SSI initially had an open injury. The one patient with a deep infection in the vancomycin-treated group had an injury that was initially open. There were no cases of superficial SSI in the vancomycin-treated group and 7 cases (8 %) of superficial SSI in the control group of 83 patients. The rate of superficial SSI was not statistically different between the two groups (*P* = 1.0). Seven (8 %) of the 83 control patients and one (10 %) of the ten vancomycin-treated patients had concomitant compartment syndrome requiring surgical release. The incidence of compartment syndrome was not statistically significant amongst the two groups (*P* = 1.0).

There was no significant difference in average time (in days) to definitive surgical treatment amongst the two groups (15.6 vs 19.8) for the control and vancomycin-treated groups, respectively (*P* = 0.149). The groups were statistically similar in terms of pre-operative temporizing management with 74/83 (89 %) of control group and 9/10 (90 %) of the vancomycin-treated patients being externally fixed prior to definitive management (*P* = 1.0). Thirty-four (41 %) of the 83 control patients underwent dual-incision operations at time of definitive fixation of their plateau and pilons, and 59 % (49/83) had a single incision. In the vancomycin-treated group, four out of ten (40 %) had single incisions and 60 % had dual incisions. The number of incisions did not differ statistically among the two groups (*P* = 1.0).

In the vancomycin-treated group, the surgical site cultures obtained from the one clinically infected patient at time of surgical irrigation and debridement showed no growth of pathogen at 6 months. Of the 14 infections in the control group, 6 yielded Methicillin-sensitive* Staphylococcus aureus* (MSSA), 4 resulted in Methicillin-resistant* Staphylococcus aureus* (MRSA), 1 grew* Staphylococcus epidermidis*, and 3 had no microbiology data available. One of the patients that grew MRSA concomitantly grew* Escherichia coli*, and one patient with MSSA cultures also grew* Enterobacter aerogenes*.

## Discussion

Even with timely preoperative IV antibiotic prophylaxis, meticulous attention to sterile technique, and less invasive surgical procedures, postoperative infections continue to occur in high-energy lower extremity fractures. Wound infections can dramatically increase health care resource utilization and further increase morbidity, especially in patients with extremity trauma. The post-operative infection rates in the lower extremity are reported to be as high as 60 % [6–11]. The objective of the present study was to investigate the efficacy of vancomycin powder in the prevention of deep SSI in high-energy lower extremity fractures—something previously not studied.

We found no significant difference in the rate of deep or superficial SSI high-energy tibia fractures when intra-wound vancomycin powder was administered. There are several possible unanswered questions with the use of local vancomycin powder that necessitate more refined study. The amount of local vancomycin necessary is unknown, and it is possible that 1 g intra-wound vancomycin does not achieve a therapeutic concentration, or does not do so for a sufficient period of time. Perhaps in such a challenging environment an antibiotic-eluting vehicle would be beneficial such that prolonged local concentrations can be maintained.

Pathogen resistance or targeting of inappropriate pathogens may be another reason for antibiotic failure. No cases of vancomycin resistance were seen in the infected patients of the control group (*n* = 14), illustrating that vancomycin resistance is not a significant issue in our region. Only one of the patients in the treatment group had an infection, and cultures from the surgical site did not grow any pathogens. It is difficult to surmise a connection between vancomycin administration and culture-negative infection given the *n* = 1.

Our results are consistent with the work of Martin et al. [19], who demonstrated no change in deep SSI in spinal deformity cases (*n* = 305), and Mohammed et al. [20] who showed no difference in SSI in the inguinal region after vascular procedures (*n* = 424) with the administration of intraoperative vancomycin. Martin et al. [19] mentions that spinal deformity patients are at higher risk for infection due to very large incisions, greater time of surgery, greater blood loss, greater exposure to instruments and increased amount of instrumentation. With regards to inefficacy of vancomycin in inguinal wounds, the authors postulated that the wound bed was not vascular enough for systemic absorption of vancomycin, which could have helped combat deep infections.

Several limitations of this study should be noted. The small sample size limits the statistical power of our study and the results must be interpreted with caution. The lack of patient randomization could have led to surgeon bias and treatment of the worst-appearing wounds with vancomycin, falsely giving the impression of vancomycin inefficacy. The incorporation of data from multiple surgeons also introduces bias. Operative time, a factor known to contribute to postoperative infections was not recorded. Additional factors, such as limited mobility, malnutrition, and various medical comorbidities, contribute to infection and were not controlled for in this study as we were unable to reliably obtain complete information due to the study’s retrospective nature.

With the high cost of managing the sequelae of SSI and the growing emphasis of the health care system on reduction of readmissions, it is important to investigate methods of reducing such complications. There is a need to find effective, cheap, and widely available methods for prevention of SSI. At a cost of approximately US $7 for 1 g vancomycin powder, it is a low-cost measure that has shown efficacy in lowering SSI rates for some surgeries, and should be investigated further [18]. Our study provides an initial look at vancomycin use in orthopaedic extremity trauma and recommends the need for further study. Basic science and larger prospective clinical studies are needed to further delineate the role of local vancomycin powder as a modality to reduce deep SSI in extremity trauma.
